# Inflammasome Targeted Therapy as Novel Treatment Option for Aortic Aneurysms and Dissections: A Systematic Review of the Preclinical Evidence

**DOI:** 10.3389/fcvm.2021.805150

**Published:** 2022-01-20

**Authors:** Markus Wortmann, Rosa Klotz, Eva Kalkum, Susanne Dihlmann, Dittmar Böckler, Andreas S. Peters

**Affiliations:** ^1^Department of Vascular and Endovascular Surgery, University Hospital Heidelberg, Heidelberg, Germany; ^2^Study Center of the German Surgical Society (SDGC), University of Heidelberg, Heidelberg, Germany; ^3^Department of General, Visceral and Transplantation Surgery, University Hospital Heidelberg, Heidelberg, Germany

**Keywords:** aortic dissection, aortic aneurysm, inflammasome, NLRP3, aortic disease

## Abstract

Both aortic aneurysm and dissection are life threatening pathologies. In the lack of a conservative medical treatment, the only therapy consists of modifying cardiovascular risk factors and either surgical or endovascular treatment. Like many other cardiovascular diseases, in particular atherosclerosis, aortic aneurysm and dissection have a strong inflammatory phenotype. Inflammasomes are part of the innate immune system. Upon stimulation they form multi protein complexes resulting mainly in activation of interleukin-1β and other cytokines. Considering the gathering evidence, that inflammasomes are decisively involved in the emergence and progression of aortic diseases, inflammasome targeted therapy provides a promising new treatment approach. A systematic review following the PRISMA guidelines on the current preclinical data regarding the potential role of inflammasome targeted drug therapy as novel treatment option for aortic aneurysms and dissections was performed. Included were all rodent models of aortic disease (aortic aneurysm and dissection) evaluating a drug therapy with direct or indirect inhibition of inflammasomes and a suitable control group with the use of the same aortic model without the inflammasome targeted therapy. Primary and secondary outcomes were incidence of aortic disease, aortic rupture, aortic related death, and the maximum aortic diameter. The literature search of MEDLINE (*via* PubMed), the Web of Science, EMBASE and the Cochrane Central Registry of Registered Trials (CENTRAL) resulted in 8,137 hits. Of these, four studies met the inclusion criteria and were therefore eligible for data analysis. In all of them, targeting of the NOD-, LRR- and pyrin domain-containing protein 3 (NLRP3) inflammasome effectively reduced the incidence of aortic disease and aortic rupture, and additionally reduced destruction of the aortic wall. Treatment strategies aiming at other inflammasomes could not be identified. In conclusion, inflammasome targeted therapies, more precisely targeting the NLRP3 inflammasome, have shown promising results in rodent models and deserve further investigation in preclinical research to potentially translate them into clinical research for the treatment of human patients with aortic disease. Regarding other inflammasomes, more preclinical research is needed to investigate their role in the pathophysiology of aortic disease.

**Protocol Registration:** PROSPERO 2021 CRD42021279893, https://www.crd.york.ac.uk/prospero/display_record.php?ID=CRD42021279893

## Introduction

**Figure 1:**
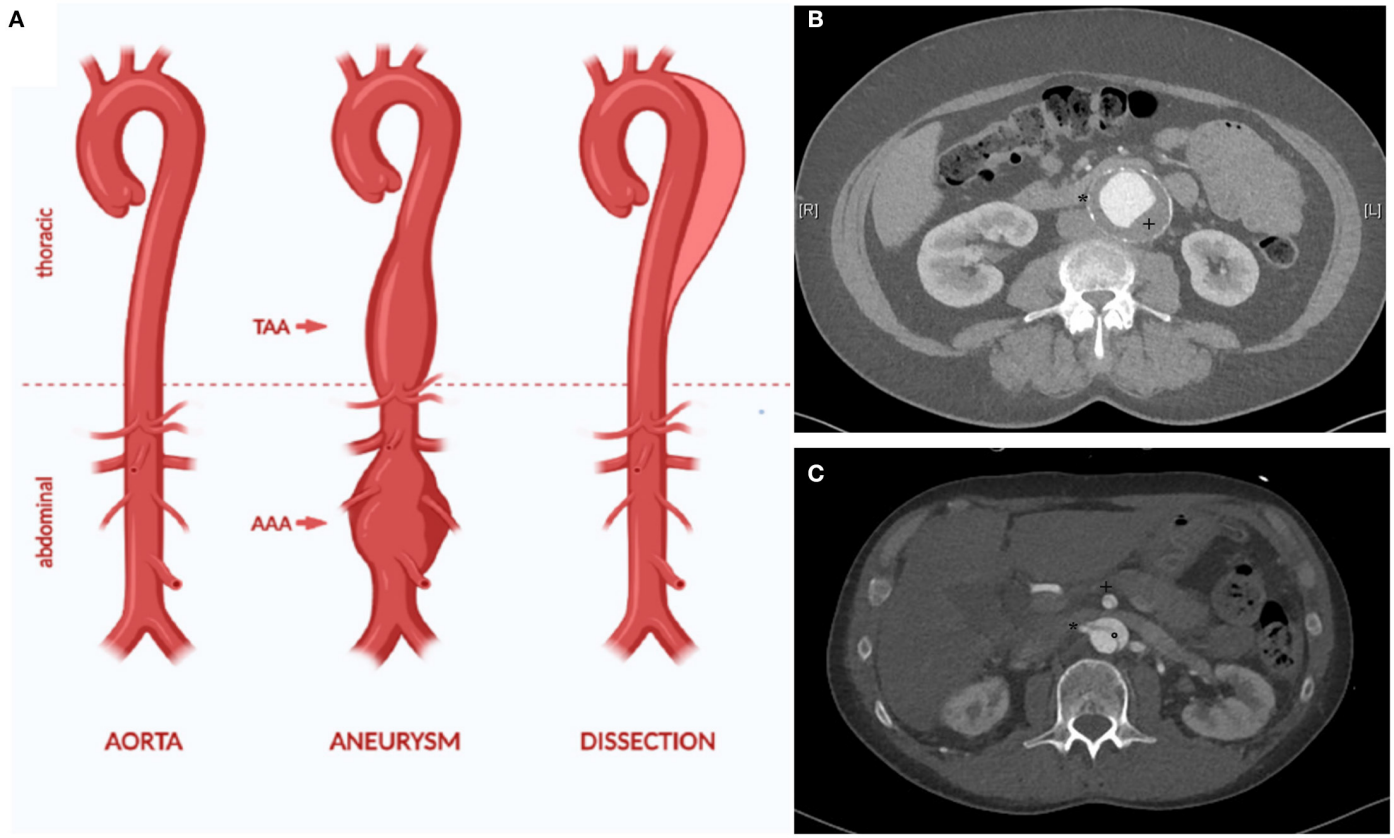
The aorta can be affected by different disease, for example aortic aneurysm and dissection **(A)**. AA is defined as dilatation of all layers of the vessel wall (true aneurysm). This can affect all parts of the aorta, for example the thoracic and the abdominal aorta (thoarcic aortic aneurysm (TAA) and abdominal aortic aneurysm (AAA). The CT angiography depicts characteristic traits of an infrarenal aneurysm with thrombus (+) and calcification of the aortic wall (^*^) **(B)**. In AD, an initial intima lesion causes separation of the aorta in a true and false lumen by a dissection membrane (°) **(C)**. Visceral arteries such as the superior mesenteric artery (+) and the right renal artery (^*^) can be affected by the dissection causing organ ischemia. Due to the reduced stability of the aortic wall, most patients with AD require surgical treatment in the long term due to chronic dilatation of the aorta [modified from (2)].

Aortic aneurysm (AA) and dissection (AD) are both life-threatening diseases (1). AA is a chronic dilatation of the whole vessel wall (Figure 1). This process silently occurs over years or even over decades, typically without causing any symptoms (1). Yet, with raising diameter, the risk of rupture increases as well. Rupture of an AA is life-threatening and still leads to death in 30 up to 85% of the affected patients, despite modern, minimal invasive endovascular treatment modalities (3, 4).

In AD, a tear in the inner wall of the aorta causes a pathological separation of the interna/media layer from the outer aortic wall (Figure 1). If the ascending aorta is affected, emergency cardiac surgery is required due to a high lethality up to 50% in the first week after diagnosis (5, 6).

A dissection of the descending thoracic and abdominal aorta is primary not as lethal but can also require emergency treatment in case of complications such as rupture or organ malperfusion caused by the dissection membrane and the complex hemodynamic alterations in dissected vessels (7). According to most guidelines, primary treatment of AD of the descending thoracic aorta (Stanford B dissection) is still conservative with close monitoring on intensive care unit and strict blood pressure control (8). After 14 days, dissections transition from an acute onset to a subacute phase for 4 weeks and afterwards into to a chronic phase. The chronic phase of AD is often characterized by a slow, but mostly steady growth of the aortic diameter due to the weakness of the dissected aortic wall. Due to this aneurysmatic expansion, most patients require surgical intervention in the course of time to prevent aortic rupture.

For both AA and AD, there is no conservative treatment besides optimizing cardiovascular risk factors, especially hypertension (9). Patients are closely monitored until a disease-specific aortic diameter is reached. To prevent aortic rupture, invasive treatment is than recommended to the patients (1). Treatment options consist of open surgical aortic repair and minimal-invasive endovascular therapy, which lately have become the preferred treatment for most aortic pathologies despite randomized clinical studies comparing it to surgical open repair (10).

All patients with AA and AD would benefit from a conservative treatment that reduces growth and hence the risk of rupture, especially elderly patients with comorbidities or patients with complex pathologies requiring excessive therapy.

Although the pathogenesis of AA and AD is still largely unknown, both diseases have a strong inflammatory phenotype (2). In other cardiovascular diseases, especially atherosclerosis, anti-inflammatory treatment strategies have been successfully established in the last years. For example, targeting interleukin-1β (IL-1β) by the monoclonal antibody canakinumab reduces the rate of recurrent cardiovascular events (11). However, anti-inflammatory therapeutic approaches for AA and AD are not yet available. In this context, inflammasomes present an interesting target for the development of pharmaceutical therapies for aortic disease.

Inflammasomes are part of the innate immune system and are not only involved in infection but also in tissue injury and repair (12). They are multiprotein complexes which consist of a sensor protein, caspases, and in some but not all cases an adapter protein connecting the two. The sensor is called pattern recognition receptor (PRR) and is eponymous for the whole inflammasome. After stimulation with either pathogen associated molecular patterns (PAMPs) or damage associated molecular patterns (DAMPs), the inflammasome is assembled and starts activating interleukins, thereby initiating an inflammatory response.

There is increasing evidence that inflammasomes are involved in the complex inflammatory processes causing emergence and progression of aortic diseases such as AA and AD [reviewed by (2, 13, 14)].

This has especially been shown for the NOD-, LRR- and pyrin domain-containing protein 3 (NLRP3) inflammasome (15). In mice with a NLRP3 knockout, the incidence of aortic aneurysm and the maximum aortic diameter was significantly reduced in model of aortic disease. This was not only applicable for NLRP3, but also the other inflammasome proteins adaptor molecule apoptosis-associated speck-like protein (Asc) and caspase-1 (Casp1) (15). Interestingly, the NLRP3 inflammasome is triggered by oxidized low-density lipoprotein and cholesterol crystals and for this reason represents an interesting link between atherosclerosis and AA/AD [reviewed in (16)].

Deficiency of another inflammasome sensor, absent in melanoma 2 (AIM2), also reduces the incidence of AA in the angiotensin II mouse model for AA (17). Regarding human samples, AIM2 is increased in peripheral leukocytes of patients with AA (18). Therefore, AIM2 states another potential inflammasome target in the pathophysiology of aortic disease. The role of the other inflammasomes in this context is largely unknown.

In summary, there is growing evidence of the involvement of inflammasomes in the pathophysiology of AA/AD. This raises the question whether targeting inflammasomes might be a potential treatment strategy in aortic disease. All cited reviews have concentrated on the pathophysiology and only marginally covered therapeutic options.

As a basis for future preclinical and potential translational research projects, a systematic review focused on the actual preclinical evidence regarding the impact of direct or indirect inhibition of inflammasomes by drug treatment as potential treatment strategy for aortic disease was performed.

## Methods

This systematic review was performed according to the recommendations of the “Preferred Reporting Items for Systematic Reviews and Meta-Analysis” (PRISMA) (19). The study was registered in the “International Prospective Register of Systematic Reviews” (PROSPERO) (CRD42021279893) in 2021 and is available upon request.

### Literature Search

A systematic literature search in the databases MEDLINE (*via* PubMed, date of search: 17.08.2021), the Web of Science (date of search: 18.08.2021), EMBASE (date of search: 05.07.2021) and the Cochrane Central Registry of Registered Trials (CENTRAL) (date of search: 05.07.2021) was performed. The search also included reference lists of relevant articles and citing references in Web of Science. The patient-intervention-comparison-outcome (PICO) scheme was used with the following specifications:

- Population (P): rodent models of aortic disease (aortic aneurysm and dissection)
- Intervention (I): direct or indirect inhibition of inflammasomes by drug treatment
- Control (C): same animal model as on the intervention group without inflammasome targeted therapy
- Outcome (O): Incidence of aortic disease, aortic rupture, aortic related death, and maximum aortic diameter

Restrictions regarding date of publication or other restrictions were not applied. The search terms used can be found in Supplement 1.

Aortic disease was defined as both AA and AD. Despite being both completely different diseases on human patients, this is typical in preclinical research since the most widely used rodent model, the angiotensin II model, depicts both anatomic and histologic features of AA and AD (20).

### Study Selection and Eligibility Criteria

All rodent models using direct or indirect, yet specific, inhibitors of inflammasomes in combination with a model for aortic aneurysm or dissection were included, independent of species, age, or sex. Studies in which no inhibitors, but only genetic modifications such as knockout or knockdown were excluded. Inhibition of nuclear factor 'kappa-light-chain-enhancer' of activated B-cells (NF-κB) was not included, since this transcription factor controls the transcription of several inflammatory proteins, among which are also inflammasomes. Inhibition of interleukin-1β (IL-1β) was also excluded, since IL-1β is activated from pro- IL-1β by inflammasomes. Yet, there are many other sources of IL-1β besides inflammasome activation. Studies without a suitable control group were excluded. The study selection process was independently performed by two reviewers (MW and ASP), who subsequently reviewed the study title, abstract and full text with respect to eligibility. In case of discrepancies, a third reviewer (SD) was involved, the article intensively discussed and decided whether it was eligible. Duplicates and non-original articles such as reviews, conference or poster abstracts, letters and comments were excluded.

### Data Extraction

Data extraction from the eligible publications was performed using a standardized form by two authors (MW and ASP) independently. Any queries and discrepancies were resolved by a third reviewer (SD). Data extracted included experimental groups, group size, species, sex, age, diet, induction of aortic disease and inflammasome targeted intervention (substance, dose, application, and duration). Primary outcome was the incidence of aortic disease (AA and AD). Secondary outcomes were aortic rupture/ aortic associated death, maximal aortic diameter and changes of the inflammation detected by immunofluorescence and protein/ mRNA levels. Additionally, general information such as title of the publication, authors, year of publication, journal, and language were collected. In case of missing data, the corresponding authors were contacted.

### Risk of Bias Assessment

The methodological quality of the included studies was assessed independently by two review authors using the SYRCLE's risk of bias tool (21). The tool included six types of bias: selection bias, performance bias, detection bias, attrition bias, reporting bias and other biases. These domains were rated as high risk of bias, low risk of bias or unclear. In addition, the CAMARADES checklist in an adapted form was used for evaluating study quality (22). In case of disagreements, the authors MW and ASP discussed the studies, and a third author (SD) was inquired.

### Strategy for Data Synthesis

Due to the expected low data homogeneity caused using different species and models for aortic disease, primarily a narrative synthesis was planned. A meta-analysis was intended to be performed for all outcome measures reported in 10 or more articles. However, the number of published articles was inadequate, and data were therefore reported as a descriptive summary.

## Results

**Figure 2:**
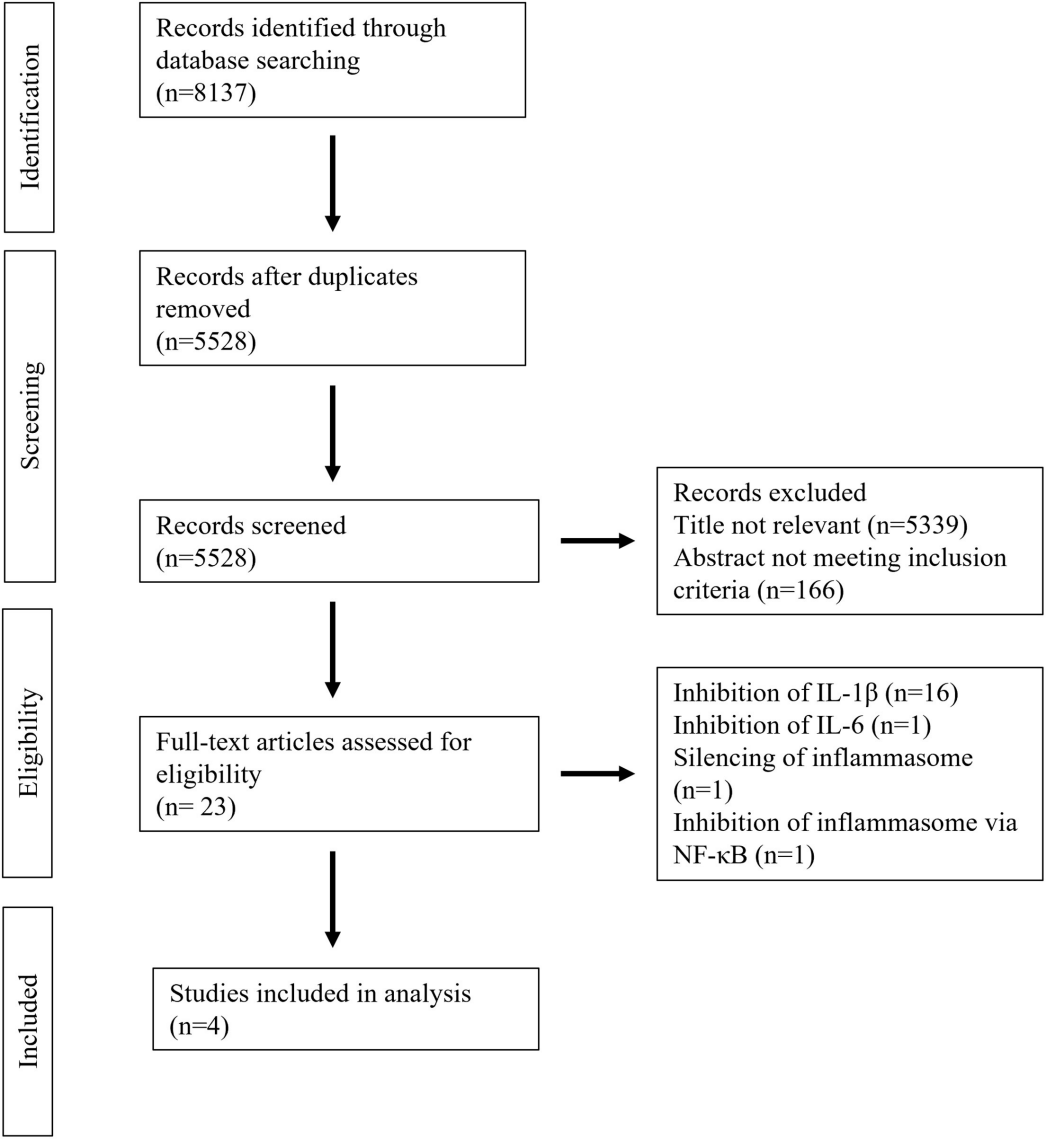
PRISMA flow chart.

A total of 8,137 studies were identified in the initial literature search, of which 8,133 were excluded due to being duplicates or not meeting the inclusion criteria (Figure 2). Finally, after full text screening four publications were included in the analysis of this systematic review (23–26). An overview of the included studies is displayed in Table 1. The studies bore a moderate risk of bias estimated by use of SYRCLE's risk of bias tool (Table 2) and showed a moderate quality assessed by the CAMARADES checklist (Table 3). Both evaluations were impeded by missing data.

**Table 1:** Characteristics and main results of the four included studies (n. r.: not reported, ^*^approximately values, exact numbers not reported).

**Reference**	**Rodent model**	**Induction of aortic disease**	**Inflammasome targeted**	**Inflammasome targeted therapy**	**Group size**	**Incidence of aortic disease**
	Species Genotype Age Sex	Substance Dose Route of application		Substance Dose Route of application Direct/indirect inhibition	Inflammasome targeted therapy vs. control, *n*	Inflammasome targeted therapy vs. control, %
Ren et al., 2020 (25)	Mouse C57Bl/6J Age: 4 weeks female and male	Ang II 2.000 ng/ min per kg Subcutaneous	NLRP3	MCC950 20 mg/kg Intraperitoneal Direct inhibition	45 vs. 50	40 vs. 86% (*p* < 0.01)
Cui et al., 2021 (26)	Rat Sprague Dawley Age: 3 weeks Sex n. r.	BAPN 0.25% Oral	NLRP3	H_2_S 100 μmol/kg Intraperitoneal Direct Inhibition	13 vs. 13	7.7 vs. 53.8%
Wu et al., 2017 (23)	Mouse C57Bl/6J Age: 4 weeks male	Ang II 2.000 ng/ min per kg Subcutaneous	NLRP3	Glyburide 5 mg/kg Oral Indirect Inhibition	36 vs. 28	75 vs. 100%^*^
Le et al., 2020 (24)	Mouse C57Bl/6J Age: 3 weeks Sex: n. r.	BAPN 1 mg/kg Oral	NLRP3	TEPP-46 5 mg/kg Intraperitoneal Indirect Inhibiton	14 vs. 14	20 vs. 85.7%^*^

**Table 2:** Evaluation of the study quality with the SYRCLE's risk of bias tool (+: no or minimal risk of bias. −: high risk of bias, ○: not reported).

**Publication**	**Selection bias**			**Performance bias**		**Detection bias**	**Attrition bias**	**Reporting bias**	**Other**
	**Sequence generation**	**Baseline characteristics**	**Allocation concealment**	**Random housing**	**Blinding**	**Blinding**	**Incomplete outcome data**	**Selective outcome reporting**	**Other sources of bias**
Ren et al., 2020 (25)	**+**	**+**	○	○	○	○	+	+	+
Cui et al., 2021 (26)	**+**	**+**	○	○	○	○	+	+	+
Wu et al., 2017 (23)	**+**	**+**	○	○	○	○	+	+	+
Le et al., 2020 (24)	**+**	**+**	○	○	○	○	+	+	+

**Table 3:** Evaluation of the study quality with a modified checklist CAMARADES checklist.

**Publication**	**1**	**2**	**3**	**4**	**5**	**6**	**7**	**8**	**Score**
Ren et al., 2020 (25)	+	+	○	○	○	○	+	+	4/8
Cui et al., 2021 (26)	+	+	+	○	○	○	+	+	5/8
Wu et al., 2017 (23)	+	+	○	○	○	+	+	+	5/8
Le et al., 2020 (24)	+	+	+	○	○	○	+	+	5/8

*1: peer reviewed publication, 2: use of a suitable animal model, 3: random allocation to treatment and control, 4: blinded induction of aortic disease, 5: blinded assessment of outcome, 6: sample size calculation, 7: compliance with animal welfare regulations, 8: statement of potential conflicts of interest, +: appropriate. -: not appropriate, ○: not reported*.

### Direct Inhibition of NLRP3 by MCC950

MCC950 is a small-molecule inhibitor of the NLRP3 inflammasome (27) which has already been shown to be beneficial in animal models for atherosclerosis and several autoinflammatory and autoimmune diseases (27, 28). It effectively decreases infiltration of inflammatory cells and NLRP3 mediated IL-1β secretion (28, 29).

Ren et al. investigated the impact of MCC950 on AA and AD in 4 weeks old male and female C57Bl/6J mice, fed a high-fat and high-cholesterol diet for 8 weeks (25). During the last 4 weeks, AA/AD was induced by continuous subcutaneous angiotensin II (AngII) application at a dose of 2.000 ng/ min per kg using osmotic mini pumps. MCC950 was daily administered by intraperitoneal injection at a dose of 20 mg/kg, control mice received PBS.

Inhibition of the NLRP3 inflammasome by MCC950 significantly reduced the incidence of AA/AD by approximately 50% (86 vs. 40%, *p* < 0.01) and rupture (18 vs. 4%, *p* = 0.04). This effect was independent of the aortic region (ascending aorta, aortic arch, descending aorta, suprarenal aorta) and of the gender of the mice. In line, MCC950 reduced aortic degeneration, elastic fiber degradation, VSMC apoptosis and VSMC contractile protein degradation. Regarding expression of inflammatory proteins, MCC950 mitigated the higher levels of procaspase-1, active caspase-1, pro-interleukin-1β and IL-1β triggered by the treatment with AngII.

The authors also showed that MCC950 diminished levels of activated matrixmetalloprotease-9 (MMP-9), which was directly cleaved by the inflammasome component caspase-1. Considering MMPs being centrally involved in aortic wall destruction, this is another encouraging effect besides reduction of inflammation [reviewed in (30)].

### Direct Inhibition of NLRP3 by Hydrogen Sulfide

Hydrogen sulfide (H_2_S) has protective effects in several cardiovascular diseases, especially atherosclerosis by its anti-oxidative and anti-inflammatory properties (31). It supresses oxidative stress induced NLRP3 (32), successfully reducing cytokine expression (33). Remarkably, H_2_S also inhibits free fatty acid and high glucose associated NLRP3 inflammasome activation which might be evolved in the inflammatory processes causing aortic disease (34, 35).

Cui et al. (26) investigated the impact of H_2_S on Aortic disease defined as AA and AD in 3 weeks old Sprague Dawley rats. Aortic disease was induced by β-aminopropionitrile (BAPN) diet (0.25% BAPN). The control group was fed a regular diet. The treatment group received additionally sodium hydrosulphide (NaHS, 100 μmol/kg) daily by intraperitoneal injection. The duration of the experiment was 6 weeks. It must be noted that the control group did not receive daily intraperitoneal injections with the carrier solution used for dissolving NaHS. Additionally, the onset of NaHS application is unclear. While in the method section NaHS seems to be administered simultaneously with BAPN, in the results section the authors stated, that NaHS was administered 1 week after BAPN.

NaHS effectively reduced the incidence of AA to 7.7% in comparison to 53.8% in the control group. Remarkably, every rat with an AA died of rupture in this model. An impact of NaHS on aortic rupture could not be demonstrated since in the NaHS treatment group only one animal developed an AA, but also died from aortic rupture. In line with the reduction of aortic disease, NaHS reduced the destruction of elastic fibers of the aortic wall. Protein expression of NLRP3, caspase-1, ASC and IL-1β was elevated in the BAPN group and ameliorated by NaHS.

Remarkably, the authors also described an interaction of NRLP3 inflammasome activation and TGF-β1 signaling which is significantly involved in the pathogenesis of aortic pathologies (36).

### Indirect Inhibition of NLRP3 by Glyburide

Glyburide is an antidiabetic drug which indirectly inhibits NLRP3 inflammasome formation by stopping potassium efflux (37).

Besides using different knockout mice, Wu et al. additionally investigated the effect of NLRP3 inhibition by glyburide using an AngII mouse model (23). Four-week-old male C57BL/6 mice received a high-fat diet for 8 weeks. In the last 4 weeks of the experiment, AA/AD was induced by subcutaneous infusion of AngII *via* an osmotic pump with a dose of 2.000 ng/min/kg. The treatment group received daily 5 mg/kg glyburide by oral gavage, the control group was given water by oral gavage.

Glyburide treatment reduced the incidence of AA/AD significantly in all aortic regions but the infrarenal aorta to an overall incidence of approximately 75% vs. 100% in untreated animals (*p* < 0.001, approximately numbers since exact values are not reported). Aortic rupture was nearly completely diminished. In line with the other studies included in this systematic review, glyburide cut the degradation of elastic fibers and adventitial remodeling.

### Indirect Inhibition of NLRP3 by the PKM2 Activator TEPP-46

Pyruvate kinase isoform M2 (PKM2) catalyses the rate-limiting step of aerobic glycolysis. PKM2 related aerobic glycolysis is closely related to IL-1β secretion *via* the NLRP3 inflammasome. Pharmaceutical activation of PKM2 by its selective activator TEPP-46 supresses NLRP-3 mediated IL-1β secretion (38, 39). Le et al. investigated the effect of an indirect inhibition of the NLRP3 inflammasome in a mouse model of aortic disease (24). AA/AD was induced in 3 weeks old C57BL/6 mice by beta-aminopropionitrile (BAPN) 1 mg/kg *via* drinking water for 4 weeks. TEPP-46 (5 mg/kg/d) was administered daily *via* intraperitoneal injection. Mice in the control group received daily intraperitoneal injections of 0.5% DMSO.

BAPN administration activated the NLRP3 inflammasome as detected by elevated mRNA and protein levels of NLRP3, ASC and caspase-1 which again was ameliorated by TEPP-46. Indirect inhibition of the NLRP3 inflammasome by TEPP-46 significantly reduced the incidence of aortic disease and aortic related mortality by more than half. Histological analysis showed that TEPP-46 reduces the loss of vascular smooth muscle cells (VSMC) and degradation of elastic fibers of the aortic wall.

## Limitations

The impact of inflammasomes in the pathophysiology of aortic disease is still vague with NLRP3 being the most intensive researched inflammasome in this context (13). Yet, there is gathering evidence that other inflammasomes- especially AIM2- also have an impact on the emergence and progression of AA and AD (14). This is the explanation for the literature search yielding only in four publications which were included in this review. While all studies use substances targeting the NLRP3 inflammasome they differ substantially with respect to the model of artic disease (AngII and BAPN) and the rodent species (mouse and rat). The low number of included studies in combination with the profound differences in the study design imped the conduction of a meta-analysis. In addition, analysis of the risk of bias and study quality by the SYRCLE's risk of bias tool and the CAMARADES checklist resulted in a moderate risk of bias and moderate study quality which both further aggravates their interpretation.

Regarding the model of aortic disease, AngII and BAPN was used in each two studies. In three studies mice were used, in one rats. Although being the most used mouse model for aortic disease, the AngII mouse model is not uncontroversial. Aneurysms in this model depict both features of AA and AD (20). Each of the different models, which have been developed for the research of aortic disease, has unique characteristics, and reflects different facets of human disease (40). In general, confirmation of the results from one model by use of another model seems rational but has not been performed for the four substances identified in this systematic review.

## Discussion

Like many cardiovascular diseases, AA and AD have a strong chronic inflammatory phenotype (41) with a significant involvement of the innate immune system, especially inflammasomes (2). While in treatment of atherosclerotic cardiovascular diseases anti-inflammatory treatment strategies have been established, so far there is no such treatment option available for treatment of AA and AD. Yet, targeting inflammasomes appears to be a promising approach. Since they are triggered by pathogen-associated molecular patterns (PAMPs) and danger-associated molecular patterns (DAMPs), inflammasomes could be a key player in the early phase of the development of AA and AD. For example, the NLRP3 inflammasome is triggered by oxidized low-density lipoprotein and cholesterol crystals (16). In animal models, it has been shown that inflammasomes are crucial for the emergence of aortic disease-so far best proven for NLRP3 (15). Additionally, there is gathering evidence for the involvement of AIM2, another inflammasome, which is triggered by free DNA, in the pathophysiology of AA (17, 18).

NLRP3 is also the best investigated inflammasome in human patients. In probes of the aortic wall sampled during open repair of infrarenal AA, the inflammasome components ASC, caspase-1, caspase-5, NLRP3 and AIM2 were histochemically identified in infiltrating immune cells in the outer media and adventitia (42, 43). NLRP3 was also higher expressed on mRNA levels compared to healthy controls (44). Another inflammasome, IFI16, was also overexpressed in human aortic aneurysm probes on mRNA and protein levels, suggesting a potential involvement in the pathophysiology of abdominal AA (45). In addition to the local findings in the aortic wall, inflammasome components are also elevated in peripheral blood mononuclear cells derived from male AA patients in comparison to healthy controls (46). With respect to thoracic AA, NRLP3, ASC, and caspase-1 expression was increased in macrophages and vascular smooth muscle cells (23). In aortic wall probes from the ascending aorta of patients with acute AD, the AIM2 inflammasome is overexpressed on protein level (47). Which impact these findings have on the evolvement or progression of aortic disease in human patients cannot be assessed now. Data on other inflammasomes or functional studies are also lacking. Due to the decrease of open surgical repair due to advances in minimal-invasive endovascular treatment option, access to probes of the aortic wall is further limited, which will make further studies even more difficult.

Besides reduction of risk factors, especially smoking cessation, and treatment of hypertension, there is no conservative treatment option for AA and AD to effectively reduce the progression rate and the risk of rupture (48, 49). Since in other cardiovascular diseases anti-inflammatory treatment concepts have proven to be effective and increasingly enter clinical patient care, a systematic review to evaluate the actual preclinical evidence for the inhibition of inflammasomes as novel treatment option for aortic disease was performed.

Four publications in which a direct or indirect inhibition of the NLRP3 inflammasome effectively reduced the incidence of aortic aneurysm and dissection and death related to aortic rupture have been identified. The effect was either indirect by TEPP-46 and glyburide or direct by MC9550 or H_2_S.

Three publications used mouse models, one a rat model of aortic disease. AA/AD was either induced with angiotensin II, with is one of the most widespread rodent models, or BAPN. Across all publications, inflammasome inhibition approximately halved the incidence of AA/AD and significantly diminished death by aortic rupture. Consistently, inflammasome inhibition prevented the aortic wall from degradation of collagen or elastic fibers. The fact that all four treatment strategies resulted in a relevant reduction of the incidence of aortic disease in independent models and species, emphasizes the potential relevance of NLRP3 targeted treatment strategies.

Besides pharmacological inhibition of NLRP3 in the four cited publications, the systematic review has not yielded any other results, especially regarding any other inflammasomes or inflammasome components. Since NLRP3 is the best researched inflammasome in the pathophysiology of aortic disease, it is not surprising that pharmaceutical inhibition of NLRP3 is also furthest advanced. The role of most other inflammasomes in the emergence and progression of AA and AD is less clear and requires further investigation. Therefore, it is not surprising, that pharmaceutical inhibition of other inflammasomes has not yet been investigated so far but might be a future research objective.

Since this systematic review focused on pharmacological treatment options which can be transitioned into patient care, genetic modifications of inflammasome components by knockout, knockdown, or silencing approaches were excluded.

Inflammasome assembly was defined being completed with activation of caspase-1. Therefore, IL-1β was regarded to be a downstream mediator of inflammasome activation (50). Yet, there is increasing preclinical evidence that targeting IL-1β signaling is effective in the treatment of aortic disease. Pharmacological inhibition of IL-1β signaling thereby is either performed by the recombinant IL-1 receptor antagonist Anakinra (51) or by a IL-1β neutralizing antibody (52). Recently, a randomized, double blind, placebo controlled clinical trial investigating ACZ885 (canakinumab), a selective, high-affinity, fully human monoclonal antibody inhibiting IL-1ß, as potential treatment for infrarenal aortic aneurysm has been aborted due to ineffectiveness (ClinicalTrials.gov Identifier: NCT02007252). This once more carves out the difficulty of translating the results of preclinical studies in rodent models to patient care. Although, IL-1β targeted treatment has been very efficient in preclinical research, it seems not to be effective in the treatment of human patients. So far it is not definite, whether the inefficiency of canakinumab in the treatment of abdominal AA is applicable for thoracic AA or AD.

Correspondingly, inhibition of NF-κB was also excluded. NF-κB is one of the most important inflammatory transcription factors, which has a proven role in aortic disease in preclinical research. NF-κB inhibition has been shown to attenuate aortic disease (53–56). Additionally, activation of NF-κB pathway induces gene expression of several inflammasomes, for example the NLRP3 and AIM2 (57). On the other hand, NF-κB is a multifaceted transcription factor that activates several genes resulting in proteins involved in inflammation, immunity, cell proliferation, differentiation, and survival, and is by far not limited to inflammasomes (58). Since this systematic review was precisely focused on inhibition of inflammasome, NF-κB inhibition was excluded, although being another interesting approach for treatment of aortic disease.

In preclinical research using rodent models, AA and AD are frequently combined with the term aortic disease. This can be attributed to the fact, that the most widely used mouse model, in which angiotensin-II is administered subcutaneously to induce aortic disease, depicts both anatomical features of AA and AD (20). In contrast, AA and AD display two completely different diseases in the term of clinical course and treatment of human patients. In the lack of a deep understanding of the complex pathophysiology of these aortic diseases involving inflammatory processes, degeneration of the connective tissue fibers and smooth muscle cells of the aortic wall, genetic factors, and biomechanical influences, the translation of preclinical results to the treatment of patients in clinical routine proves to be difficult. This is not only due to the lack of an ideal rodent model depicting all features of human aortic disease. A lately performed meta-analysis of interventions to slow the progression of aortic aneurysm in mouse models resulted in 30 studies with possible drug-based therapies. Yet, none of these therapies has shown be effective in clinical trials so far (59).

Regarding the potential use in clinical trials, glyburide, also called glibenclamide, is an oral antidiabetic drug which is widely used in the treatment of type II diabetes (60). This would facilitate clinical trials using glyburide as potential treatment option for aortic aneurysm if further preclinical studies confirm its therapeutic value. With respect to H_2_S, there are several newly developed H_2_S donors which have been examined in early clinical studies (61). Additionally, there are several established and widely used drugs containing sulfur moieties, which have been found to release H_2_S *in vivo*, for example anti-hypertensive drugs such as ACE inhibitors (61). Yet it is unclear, what the contribution of H_2_S to the overall effect of the respective drug is or whether it is relevant at all. In summary, more preclinical research is needed to further evaluate the potential beneficial effects of H_2_S donors in aortic disease. Both TEPP-46 and MC9550 lack clinical data, which would make its use in clinical trials for the treatment of AA and AD more difficult.

In conclusion, inflammasome targeted therapy is a promising research issue in AA and AD in preclinical. This systematic review has identified four different substances which directly or indirectly inhibit the NLRP3 inflammasome, which is the most widely researches inflammasome in aortic disease. Despite rising evidence for the involvement of other inflammasomes, especially the AIM2 inflammasome, there are no targeted treatments for other inflammasomes published so far. Even considering the challenges of translating preclinical results to patient care in this field, inflammasome targeted therapies might be a promising therapeutic approach in the treatment of patients with aortic diseases. Yet, at the present stage further preclinical and translational research is required to conclusively evaluate the potential benefit on inflammasome targeted therapies in AA and AD.

## Data Availability Statement

The original contributions presented in the study are included in the article/Supplementary Material, further inquiries can be directed to the corresponding author.

## Author Contributions

MW, RK, EK, and DB contributed to the design of the systematic review. EK performed the search of literature in the databases. MW and AP performed the study selection and data extraction. MW, AP, and SD wrote the manuscript. All authors have reviewed the manuscript and consented in its publication.

## Funding

This work was supported with a research grant of the Heidelberger Stiftung Chirurgie (2018/257) to MW.

## Conflict of Interest

The authors declare that the research was conducted in the absence of any commercial or financial relationships that could be construed as a potential conflict of interest.

## Publisher's Note

All claims expressed in this article are solely those of the authors and do not necessarily represent those of their affiliated organizations, or those of the publisher, the editors and the reviewers. Any product that may be evaluated in this article, or claim that may be made by its manufacturer, is not guaranteed or endorsed by the publisher.
