# Supplementary material for: Inflammasome Targeted Therapy as Novel Treatment Option for Aortic Aneurysms and Dissections: A Systematic Review of the Preclinical Evidence
Source: Front Cardiovasc Med. 2022 Jan 20;8:805150. doi: 10.3389/fcvm.2021.805150 (PMC8811141; doi:10.3389/fcvm.2021.805150)
Supplement: Supplementary file 1 [file Data_Sheet_1.DOCX]

**Supplement 1**

**Inflammasome targeted therapy as novel treatment option for aortic aneurysms and dissections: a systematic review of the preclinical evidence**

**Wortmann M, Klotz R, Kalkum E, Dihlmann S, Böckler D, Peters AS**

**Search terms used in the literature research on different platforms:**

**PubMed:**

mouse*[tiab] OR mice*[tiab] OR murin*[tiab] OR mus[tiab] OR animal*[tiab] OR rodent*[tiab] OR “experimental model” [tiab] OR rabbit*[tiab] OR rat[tiab] OR rats[tiab] OR hamster[tiab] OR "Guinea pig"[tiab] OR "Rodentia"[Mesh] OR "Animals"[Mesh]

AND

("Inflammasomes"[Mesh] OR inflammas*[tiab] OR Caspase-1[tiab] OR "Caspase 1"[Mesh] OR NLRP1[tiab] OR "NALP1 protein, mouse" [Supplementary Concept] OR NLRP3[tiab] OR "Nlrp3 protein, mouse" [Supplementary Concept] OR NLRP6[tiab] OR "Nod-like receptor pyrin domain-containing protein 6, mouse" [Supplementary Concept] OR NLRP7[tiab] OR "NLRP7 protein, rat" [Supplementary Concept] OR NLRP12[tiab] OR "NLRP12 protein, mouse" [Supplementary Concept] OR NLRC4[tiab] OR "NLRC4 protein, rat"[Supplementary Concept] OR IPAF[tiab] OR "Ipaf protein, mouse" [Supplementary Concept] OR AIM2[tiab] OR "Aim2 protein, mouse" [Supplementary Concept] OR "AIM2 protein, rat" [Supplementary Concept] OR IL-1β[tiab] OR Interleukin-1beta[tiab] OR "Interleukin-1beta"[Mesh] OR IL-18[tiab] OR Interleukin-18[tiab] OR "Interleukin-18"[Mesh] OR Pyrin[tiab] OR "Pyrin"[Mesh] OR Caspase 11[tiab] OR "Casp4 protein, rat" [Supplementary Concept] OR "Casp4 protein, mouse" [Supplementary Concept] OR IFI16[tiab] OR "Ifi16 protein, mouse" [Supplementary Concept] OR "AIM2-like receptors"[tiab] OR ALR[tiab] OR "Gasdermin D protein"[tiab] OR "GSDMD protein"[tiab] OR "Gsdmd protein, mouse"[Supplementary Concept] OR "Pyrin and HIN domain"[tiab] OR "PYHIN protein"[tiab] OR "PYHIN1 protein, mouse"[Supplementary Concept] OR "nucleotide-binding and oligomerization domain"[tiab] OR "NOD-like receptors"[tiab] OR "Absent in melanoma 2"[tiab] OR "Interferon-inducible protein 16"[tiab] OR "Apoptosis speck-like-protein"[tiab] OR ASC[tiab] OR Interleukin-1[tiab] OR IL-1[tiab] OR Caspase-4[tiab] OR Caspase-5[tiab])

AND

"Aortic Aneurysm"[Mesh] OR "Aneurysm, Dissecting"[Mesh] OR "Aortic Rupture"[Mesh] OR "Aorta"[Mesh] OR aort*[tiab] OR "Aortic Diseases"[Mesh] OR "Aneurysm"[Mesh] OR "Dissection"[Mesh] OR aneur*[tiab] OR dissect*[tiab]

**Web of Science:**

TS = (mouse* OR mice* OR murin* OR mus OR animal* OR rodent* OR “experimental model” OR rabbit* OR rat OR rats OR hamster OR "Guinea pig")

AND

TS = (inflammas* OR Caspase-1 OR NLRP1 OR NLRP3 OR NLRP6 OR NLRP7 OR NLRP12 OR NLRC4 OR IPAF OR AIM2 OR IL-1β OR Interleukin-1beta OR IL-18 OR Interleukin-18 OR Pyrin OR Caspase 11 OR IFI16 OR "AIM2-like receptors" OR ALR OR "Gasdermin D protein" OR "GSDMD protein" OR "Pyrin and HIN domain" OR "PYHIN protein" OR "nucleotide-binding and oligomerization domain" OR "NOD-like receptors" OR "Absent in melanoma 2" OR "Interferon-inducible protein 16" OR "Apoptosis speck-like-protein" OR ASC OR Interleukin-1 OR IL-1 OR Caspase-4 OR Caspase-5)

AND

TS = ("Aortic Rupture" OR aorta OR "Aortic Diseases" OR aneur* OR dissect*)

**Central**

(mouse* OR mice* OR murin* OR mus OR animal* OR rodent* OR “experimental model” OR rabbit* OR rat OR rats OR hamster OR "Guinea pig"):ti,ab,kw

OR MeSH descriptor: [Rodentia] explode all trees

AND

MeSH descriptor: [Inflammasomes] explode all trees

MeSH descriptor: [Caspase 1] explode all trees

MeSH descriptor: [Interleukin-18] explode all trees

MeSH descriptor: [Pyrin] explode all trees

MeSH descriptor: [NLR Family, Pyrin Domain-Containing 3 Protein] explode all trees

MeSH descriptor: [Interleukin-1beta] explode all trees

MeSH descriptor: [NLR Proteins] explode all trees

MeSH descriptor: [Interleukin-1] explode all trees

OR (inflammas* OR Caspase-1 OR NLRP1 OR NLRP3 OR NLRP6 OR NLRP7 OR NLRP12 OR NLRC4 OR IPAF OR AIM2 OR IL-1β OR Interleukin-1beta OR IL-18 OR Interleukin-18 OR Pyrin OR Caspase 11 OR IFI16 OR "AIM2-like receptors" OR ALR OR "Gasdermin D protein" OR "GSDMD protein" OR "Pyrin and HIN domain" OR "PYHIN protein" OR "nucleotide-binding and oligomerization domain" OR "NOD-like receptors" OR "Absent in melanoma 2" OR "Interferon-inducible protein 16" OR "Apoptosis speck-like-protein" OR ASC OR Interleukin-1 OR IL-1 OR Caspase-4 OR Caspase-5):ti,ab,kw

AND

MeSH descriptor: ["Aortic Aneurysm"] explode all trees

MeSH descriptor: ["Aneurysm, Dissecting"] explode all trees

MeSH descriptor: ["Aortic Rupture"] explode all trees

MeSH descriptor: [Aorta] explode all trees

MeSH descriptor: ["Aortic Diseases"] explode all trees

MeSH descriptor: [Aneurysm] explode all trees

MeSH descriptor: [Dissection] explode all trees

OR (aort* OR aneur* OR dissect*)

**EMBASE**

(mouse* OR mice* OR murin* OR mus OR animal* OR rodent* OR “experimental model” OR rabbit* OR rat OR rats OR hamster OR "Guinea pig")

OR emb(mouse OR rat)

AND

emb (Inflammasomes OR “Caspase 1” OR NLRP1 OR NALP1 OR NLRP3 OR NLRP6 OR NLRP7 OR NLRP12 OR NLRC4 OR IPAF OR AIM2 OR IL-1β OR Interleukin-1beta OR IL-18 OR Interleukin-18 OR Pyrin OR “Caspase 11” OR Casp4 OR IFI16 OR "AIM2-like receptors" OR ALR OR "Gasdermin D protein" OR GSDMD OR PYHIN OR "nucleotide-binding and oligomerization domain" OR "NOD-like receptors" OR "Absent in melanoma 2" OR "Interferon-inducible protein 16" OR "Apoptosis speck-like-protein" OR ASC OR Interleukin-1 OR IL-1 OR Caspase-4 OR Caspase-5)

OR (inflammas* OR Caspase-1 OR NLRP1 OR NALP1 OR NLRP3 OR NLRP6 OR "Nod-like receptor pyrin domain-containing protein 6" OR NLRP7 OR NLRP12 OR NLRC4 OR ASC OR IPAF OR AIM2 OR IL-1β OR Interleukin-1beta OR IL-18 OR Interleukin-18 OR Pyrin OR Caspase 11 OR "Casp4 protein" OR IFI16 OR ALR OR "Gasdermin D protein" OR "GSDMD protein" OR "PYHIN protein" OR "nucleotide-binding and oligomerization domain" OR "NOD-like receptors" OR "Absent in melanoma 2" OR "Interferon-inducible protein 16" OR "Apoptosis speck-like-protein" OR Interleukin-1 OR IL-1 OR Caspase-4 OR Caspase-5)

AND

aort* OR aneur* OR dissect*

OR emb("Aortic Aneurysm" OR "Aortic Rupture" OR Aorta OR "Aortic Diseases" OR Aneurysm OR Dissection)
